# Potential fluoride exposure from selected food crops grown in high fluoride soils in the Makueni County, south-eastern Kenya

**DOI:** 10.1007/s10653-022-01240-w

**Published:** 2022-03-09

**Authors:** Patrick Kirita Gevera, Mark Cave, Kim Dowling, Peter Gikuma-Njuru, Hassina Mouri

**Affiliations:** 1 Department of Geology, University of Johannesburg, P.O. Box 524, Kingsway, Auckland Park, 2006 South Africa; 2 British Geological Survey, Nottingham, NG12 5GG UK; 3 School of Science, STEM College, RMIT University, Melbourne, Australia; 4 Department of Environmental Science and Land Resources Management, South Eastern Kenya University, P.O. Box 170-90200, Kitui, Kenya

**Keywords:** Makueni County, Food security, Fluoride in food crops, Soil quality, Water-soluble fluoride

## Abstract

Makueni County, located in south-eastern Kenya, faces challenges such as limited potable water and restricted food supplies as the result of semi-aridity. High fluoride (F) concentrations have been reported in drinking water with resultant dental fluorosis affecting the local population. To determine the potential F exposure through the consumption of food crops grown in the area, F concentration was assessed in the main five locally grown and consumed crops. Additionally, the water-soluble F fraction was determined from 30 soil samples with mineralogical determination of 20 samples. Mean F concentration in the food crops was in the order; 700, 288, 71.2, 36.6, and 29 mg/kg in kale, cowpeas leaves, green grams, cowpeas (legume portion), and maize, respectively. The F concentration in farm soils ranged from 0 to 3.47 mg/kg (mean of 0.87 mg/kg) and showed a significant strong positive correlation (*p* = 0.03, *r* = 0.89) with F values in the crops. Apatite, muscovite, and biotite were identified as the F-rich minerals present. While considering two hypothetical F absorption fractions (75 and 100%), the estimated average daily dose (EADD) of F from consuming the crops ranged between 0.004 and 65.17 mg/kg/day where the highest values were from the vegetables. Most of these values were higher than the F reference dose (RfD) of 0.06 mg/kg. The estimated EADD values of several hypothetical meals prepared from the analyzed crops revealed that steamed kale and maize porridge pose the highest health risk of F associated diseases to the local population, whereas boiled cowpeas pose no health risk. Children, due to their higher daily energy requirement and low body weight, were the most vulnerable group at risk of high daily F intake relative to the RfD. These results suggest that consumption of the analyzed food crops in Makueni County may significantly contribute to F related diseases in the local population. This creates a food security issue for the area because of the potential health risks associated with these crops which are highly relied upon in the semi-arid area with a limited selection of food crops available and viable to grow.

## Introduction

Food security and good health are among the United Nations sustainable development goals (UN, 2018). Attaining these goals means ensuring the provision of sufficient nutritious food to the population that is free from contaminants (Omisore, 2018). In Kenyan rural areas, about 70% of the consumed food is produced locally (Mohajan, 2014; Mungai, 2014) hence, the local environment directly influences the populations health status. Globally, there is evidence and awareness of the adverse health issues from chemically contaminated food crops (Rai et al., 2019). Among these elements is fluoride (F), a widely known natural groundwater contaminant, whose health risks in food crops are not well documented (Gevera et al., 2018; Memba et al., 2021; Rizzu et al., 2021).

F in crops grown in contaminated soils and/or irrigated with high F water could exacerbate its health effects on the consumers (Xie et al., 2007). Fluoride accumulation in plants is controlled by factors such as plant species and the availability of soluble F in farm soils (Bhattacharya et al., 2017). Weathering of F-rich rocks releases F in soils where it is absorbed by the food crops through root transfer (Rai et al., 2019). Its mobility is governed by factors including soil particle size, pH, the presence of organic matter, and clays (Abugri & Pelig-Ba, 2011; Nyika et al., 2020). Understanding these factors is crucial in controlling F dose received by local populations to improve the health status in high F regions.

Makueni County a semi-arid region in south-eastern Kenya, has low and unreliable rainfall patterns with limited surface water resulting in high dependence on groundwater sources for domestic and agricultural use (Gevera et al., 2020; Mailu, 1994; Ng’ang’a et al., 2018). F levels up to 7.17 mg/L in these water sources as well as cases of dental fluorosis have been reported in the area (e.g., Gevera et al., 2020).

Despite the concerns about the high F content in groundwater used for irrigation in the study area, to the best of our knowledge, this is the first study investigating the F concentrations in these food crops, in the farm soils where the crops are grown, as well as the potential health risks from consuming these foods. The purpose of this paper is to address this significant knowledge gap by linking F concentrations in farm soil to the mineralogy of the soil and some selected commonly grown and consumed food crops in the area. The exposure dose and health risk associated with F in the food samples were also calculated using standard methodologies.

## Study area

### Location, population, and climate

**Fig. 1:**
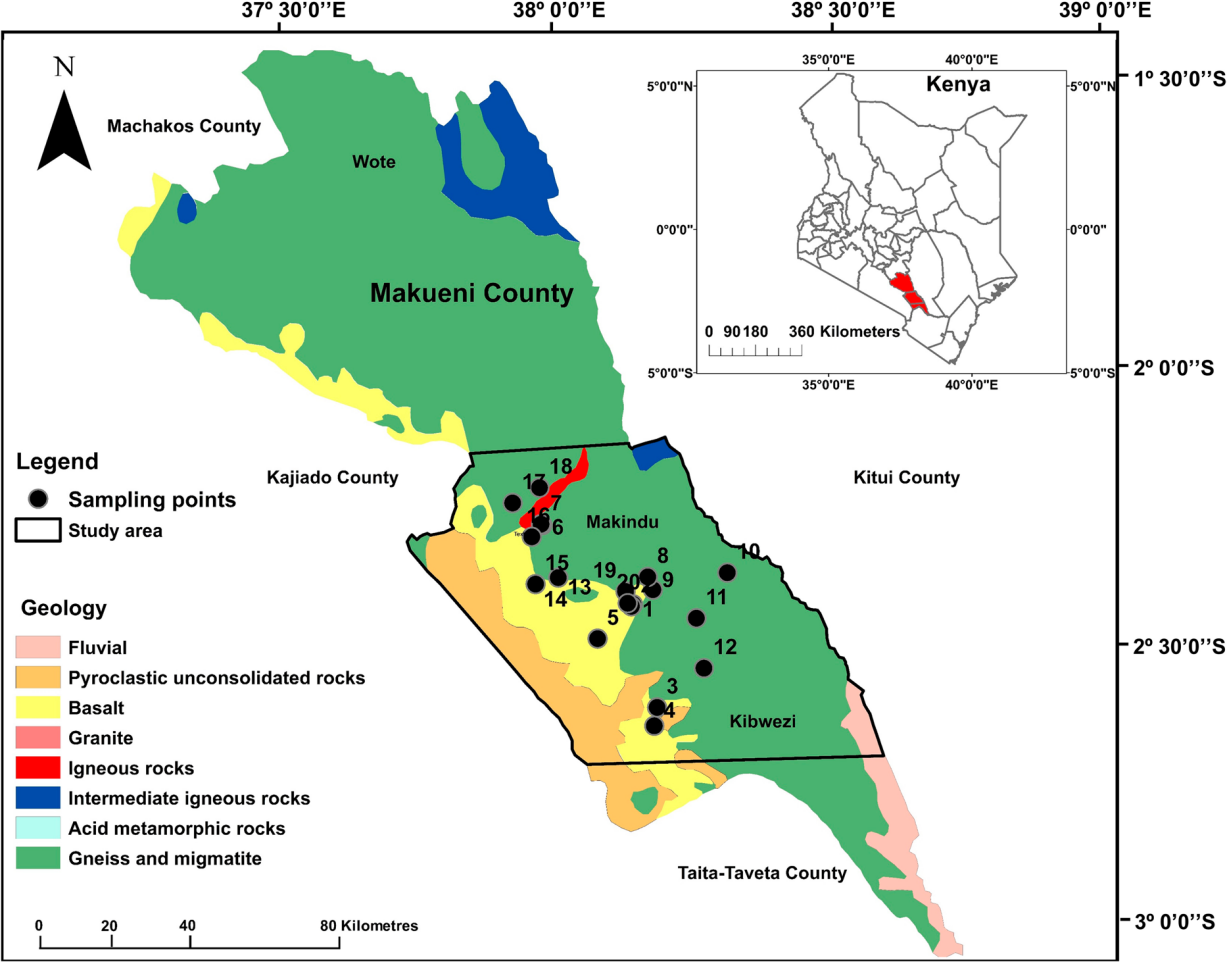
The geological map of Makueni County showing the Makindu-Kibwezi area, bounded in black, where soil and food crop samples were collected close to sampled water sources (black dots). Adapted from (Gevera et al., 2020)

Makueni County is located in the south-eastern region of Kenya and borders three counties, Machakos, Kajiado, Taita-Taveta, and Kitui (Fig. 1). The county’s population is approximately 884,500 where most of the people live in a predominately rural setting (Kenya National Bureau of Statistics, 2010; Njonjo, 2013). This paper reports a study conducted in the county’s southern region covering the Makindu-Kibwezi area as demarcated by the black outline in Fig. 1. The area was recently assessed for its drinking water quality and the health implications of F and salinity on the local population (Gevera et al., 2020, 2022 under review).

Unreliable rainy seasons produce an average annual rainfall range between 200 and 700 mm, (Muema, 2018), where planting usually occurs during the two rainy seasons in March–April and November–December. Irrigation is practiced in farms close to rivers, springs, and in few farms that use borehole water. Common food crops grown and consumed in the area include leaf and legume cowpeas (*Vigna unguiculata*), beans (*Phaseolus vulgaris*), maize (*Zea mays*), green grams (*Vigna radiata*), kale (*Brassica oleracea var. viridis*), cabbage (*Brassica oleracea var. capitata*), mangoes (*Mangifera indica*), and watermelons (*Citrullus lanatus*).

## Geology

Makueni County falls under the metamorphic Mozambique Mobile Belt (MMB) in Kenya (Nyamai et al., 2003; Saggerson, 1963). The geology of the area is dominantly metasediments composed of biotite, muscovite, and hornblende schists and gneisses as well as granitoid gneisses and granites, which are overlain by Pleistocene basalts flows and volcanic cones in the southern region (Dodson, 1953; Nyamai et al., 2003) as shown in Fig. 1. These rocks are rich in biotite, muscovite, and apatite which are potential F-hosting minerals. Soils in the area range texturally from clayey, sandy-clay loam to sandy-clay and have a general porous massive structure (Mora-Vallejo et al., 2008).

## Materials and methods

### Sampling, preparation, and analysis

All collected samples (soil and food crops) were transported to South Africa where preparation and analysis were conducted at the University of Johannesburg, Geology Department, and Agricultural Research Council (ARC) laboratories in Pretoria.

### Sample collection and preparation

**Fig. 2:**
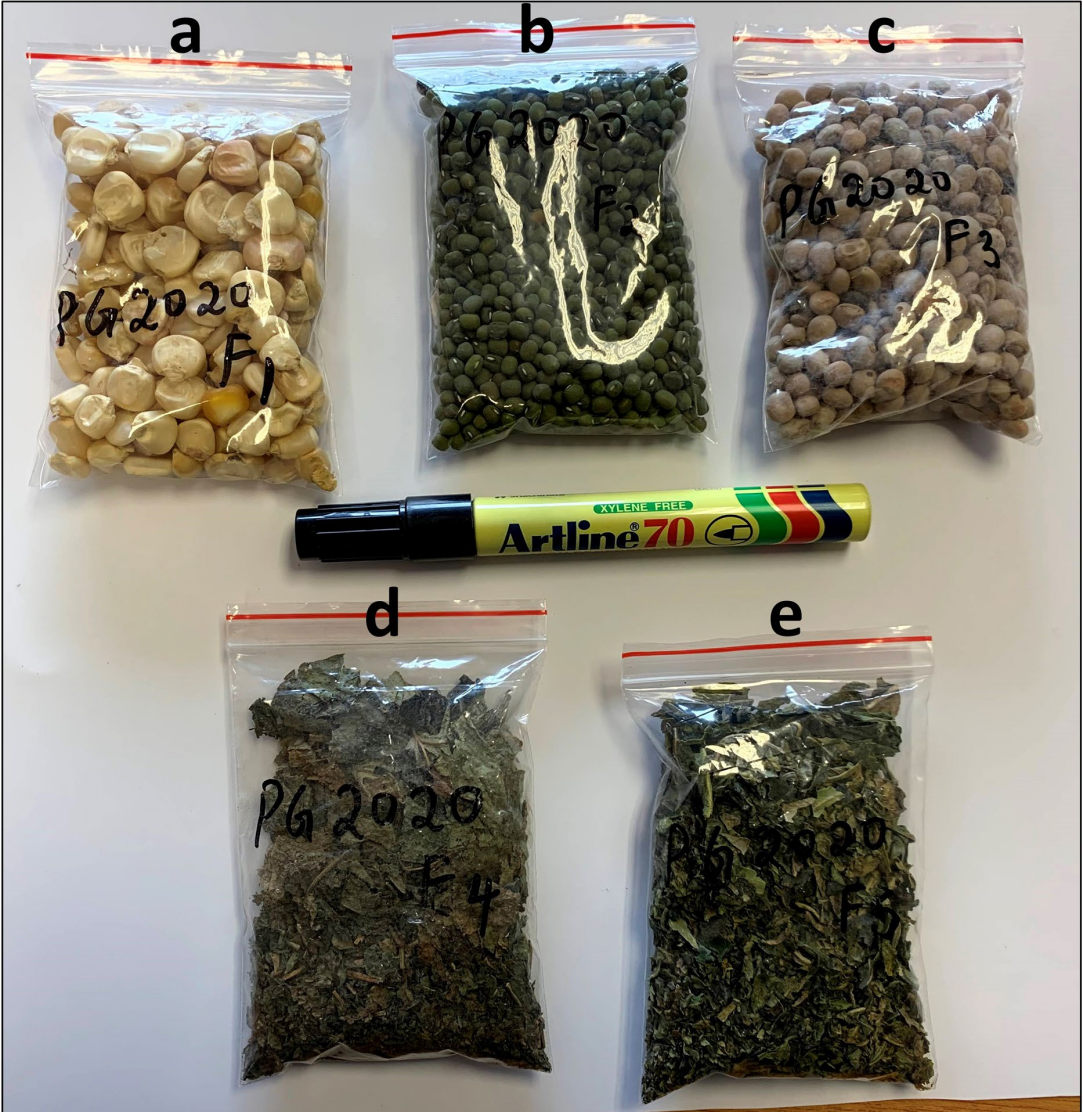
Samples of maize **a** green grams **b** cowpeas **c** grains and leaves of cowpeas leaves **d** and kale **e** from Makueni County that were collected for F analysis

*Food samples* Five commonly grown and consumed food crop samples, including three grain-based (maize, green grams, and cowpeas) and two vegetables (kale and cowpeas leaves), were collected in five farms in the southern region of Makueni County (Fig. 1) in January 2020. The samples include the edible parts of the crops (grains and leaves) as shown in Fig. 2. Grain and leaves crop samples were collected to determine how F concentrates in these two food crop types commonly consumed in the area. The samples were collected close to previously sampled drinking water sources which showed elevated F levels up to 7.17 mg/l (Gevera et al., 2020). For each food crop, samples were collected from four spots in the specific farm and mixed to attain a representative sample size of 100 g. The food samples were then stored in plastic bags kept refrigerated until they were ready to submit to the ARC laboratories for analysis. At the laboratory, the samples were crushed and sieved through a 1.4 mm mesh size to attain uniformity and oven-dried for two hours.

*Soil samples* 30 soil samples were collected in 30 farms, including those where food crops were sampled from, in December 2018 (20 samples) and January 2020 (10 samples). Farms sampled did not use fertilizers. Samples were collected from four locations in each farm and thoroughly mixed to get a homogenous representative sample size of approximately 500 mg. Each sample was collected by digging a 30 cm hole after the top layer of about 3 cm was removed to prevent the collection of plant material and other debris. The sampling depth chosen was within the root zone of the sampled food crops. Sampling was done distal to homesteads, buildings, and roads to avoid any anthropogenic contamination. The samples were kept in tightly closed plastic bags and later air-dried overnight.

A portion of the soil samples collected during the first field visit (*n* = 20) was prepared for XRD analysis at the Spectrum facility of the Faculty of Science at the University of Johannesburg to determine the presence of F-rich minerals in the farm soil which would warrant further F analyses in the soil samples. The samples were air-dried overnight and then crushed using an auger milling machine for three minutes to attain a 100 μm sieve size before analyzed. Subsequently, after ten more samples were collected during the second field visit, all the soil samples (*n* = 30) were submitted to the ARC laboratories for the analysis for water-soluble F concentration.

### F analysis

At the ARC laboratories, the five food crops (one sample for each crop) and 30 soil samples were analyzed for F concentration. For both soil and food crop samples, the partial leaching method was used to dissolve the water-soluble F from the soil. The concentrations of F were determined using the EPA Method 300.0 (Pfaff, 1993).

The 1:10 (sample: water) ratio was used for partial dissolution. Five grams of each sample was weighed into a glass beaker and mixed with 50 ml of deionized water. The mixture was shaken in a mechanical shaker for 30 min to liberate the water-soluble F fraction in the samples. The mixture was then filtered into a volumetric flask, and the concentration of F was determined, in triplicate, using a Dionex ICS 1600 ion chromatograph. The standard solution was prepared by dissolving 2.21 g of sodium fluoride (NaF) in deionized water and diluted to 1 L.

### Mineralogical analysis

The semi-quantitative abundance of minerals in 20 samples of farm soil material was determined using the Panalytical X’Pert powder diffractometer. The milled soil samples were pressed into sample holders and loaded into the XRD instrument. Operational conditions were: Cu-*K*_α_ radiation with the generator settings of 40 mA and 40 kV, and scanning angle range of 4.01 2 – 89.98 2θ. The data were collected by the *X*’Pert-Pro data collection software and interpreted using the *X*’Pert HighScore Plus software package.

### Data analysis

*F transfer factor (TF)* The concentration of F in the selected food crops is correlated to the water-soluble F values in the farm soil where the crops were grown to determine the transfer factor. The determination of the transfer of potentially harmful elements such as F from the soil to food crops is essential to advise what crops are suitable for cultivation in these specific soils (Gupta & Banerjee, 2011). The TF values were calculated using the following equation, (Rai et al., 2019).

TF = Concentration of element in crop-vegetable (mg/kg)/ concentration of element in soil (mg/kg).

*F exposure dose* F exposure dose associated with consumption of the analyzed food crops is essential to establish the health risk to the local population. The F estimated average daily dose (EADD) linked to the consumption of the five analyzed food crops was calculated from the following equation, (Rizzu et al., 2020):

EADD_F_ = C x DCI x FEC x AF/E.

Where; C is the concentration (mg/kg) of F in the different food crops analyzed.

DCI is the daily calorie intake per person: the values of 90 kcal per day/kg of body weight which is the recommended energy consumption for children below one year and 24 kcal per day/kg of body weight recommended for adolescents and adults (FAO, 2004; Rizzu et al., 2020) were used.

FEC is the food energy contribution of each analyzed food crop. The values of 47% for cereals (maize), 2% for grain legumes, and 3% for vegetables were used, based on their energy contribution to the diet of a study population in Kitui County, north of Makueni (Hansen et al., 2011).

AF is the absorption fraction of F in in the gastro-intestinal tract, where the hypothetical rates of 75 and 100% (Bhattacharya et al., 2017; Rizzu et al., 2020) is adopted. These are the two limits at which F ingested in food is absorbed in the body (Rizzu et al., 2020).

E is the energy contribution of each specific analyzed food crop (Kcal/Kg). The energy contribution (*E*) of most locally consumed foods in Kenya is reported by FAO and the Government of Kenya (FAO & Government of Kenya, 2018). The E values used (Kcal/kg) for the analyzed food crops (raw and dry) were 3450, 3110, 3130, 30, and 29 for maize, green grams, cowpeas legumes, cowpeas leaves, and kale, respectively.

*Calculation of the F EADD values of cooked meals* Given that the analyzed food crops are prepared and consumed in several forms by the population in the Makueni County, we determined the F dose associated with different meals commonly consumed in the area. This is because, meals prepared from the same crop or ingredient have different energy values (*E*), depending on the cooking method, which will in turn impact the F EADD value. A study by FAO and the Government of Kenya (FAO & Government of Kenya, 2018) reports the metabolizable energy values (*E*) of commonly consumed meals in Kenya.

In the study, the nutrition values of 100 g dry weight of several foods cooked in different ways were determined (FAO & Government of Kenya, 2018). The *E* values from cooking methods including boiling, steaming, and stir frying (for vegetables) we used to determine the EADD of the analyzed food crops in this study. The methods used in preparing the meals include boiling and draining the water after the meals were cooked, then the nutritional value of the dry cooked meals were determined (FAO & Government of Kenya, 2018).

The energy (*E*) values in used for the EADD calculations in the current study, as obtained from the FAO and the Government of Kenya (FAO & Government of Kenya, 2018) study are; 1460 for boiled maize, 1410 and 520 for whole maize floor meal (*ugali*) and porridge, 1160 and 1090 for boiled and stewed green grams, 1170 and 1320 for dry boiled and fresh boiled cowpeas, 280 for cowpeas leaves, and 1150, 1050, and 540 for boiled, steamed, and stir-fried kale as obtained from the Kenyan food nutritional data (FAO & Government of Kenya, 2018).

*F health risk assessment* The hazard quotient (HQ) associated with the five analyzed food crops was calculated to determine the individual health risk of food dietary F intake using the following formula (Rizzu et al., 2020),

HQ = EADD/RfD.

Where; RfD is the reference dose for humans associated with the ‘no observable adverse effect level’ (NOAEL) for F, which is set as 0.06 mg/kg by USEPA (US-EPA, 2003). Then, the cumulative hazard index (HI) was evaluated by adding the HQ of all the five food crops analyzed.

HI = HQmaize + HQcowpeas + HQgreen grams + HQkale + HQcowpeas leaves.

It should be noted that, the calculated HI is an estimate of only the five analyzed food crops and addition of other F sources into the diet, such as drinking water and beverages, would increase the risk.

*Statistical analyses* Several statistical analyses were employed to determine the relationships between the different variables in the dataset, these include:

Bar graphs used to visually present F concentrations in different food crops, Spearman’s correlation analysis was used to determine the strength (*r* value) and significance (*p* value) of correlation between variable such as F concentrations in farm soils and food crops, Linear correlation graphs were used to correlate the F EADD and the daily energy intake per Kg of body weight of the analyzed food crops to determine the possible health effects of F due to the food consumption. Microsoft Excel and IBM SPSS Statistics 25 were used to determine the statistical characteristics of the data.

## Results

### F concentration in food crops

**Fig. 3:**
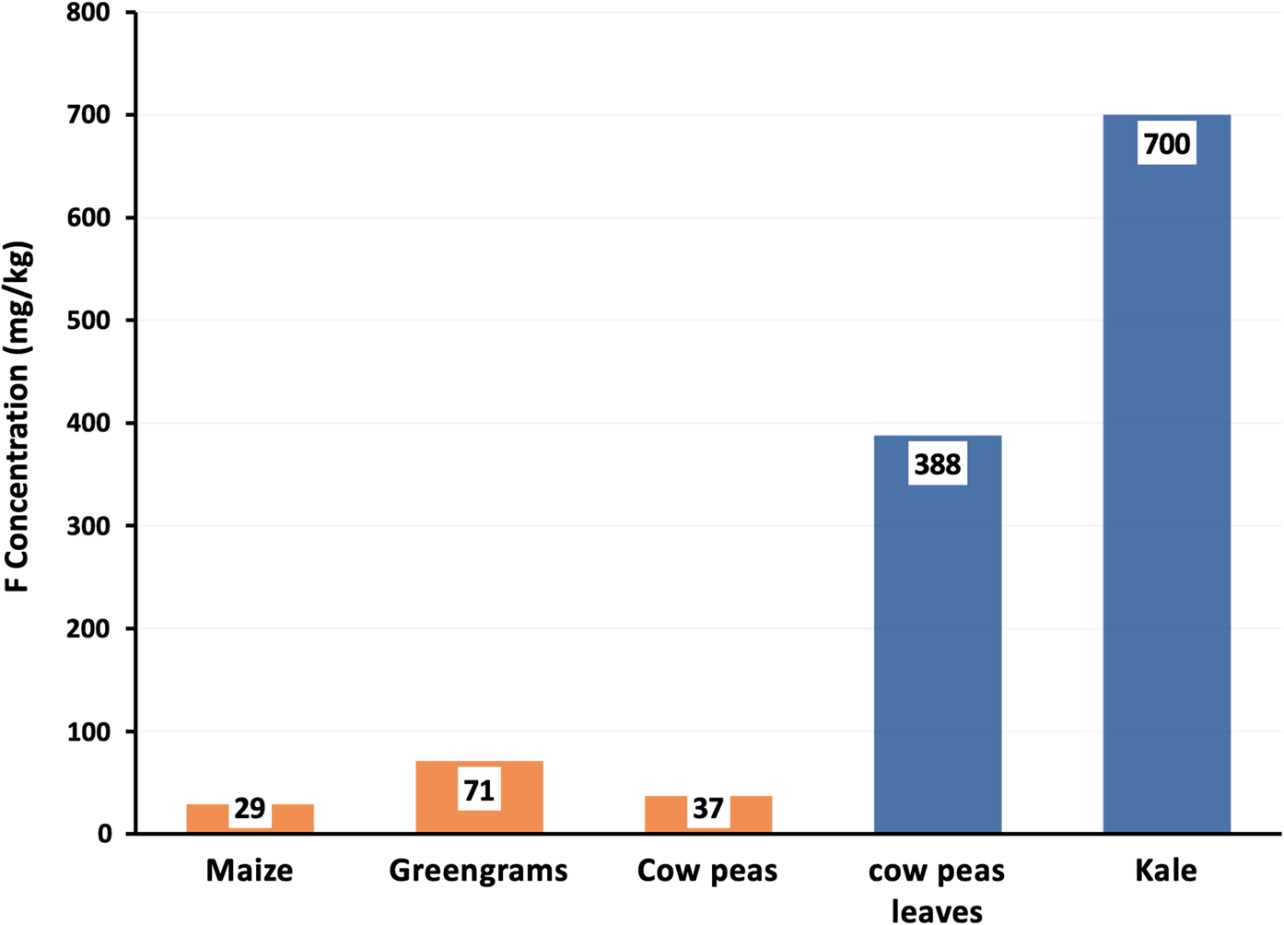
Fluoride concentration in selected five food crops including grains (maize, green grams, and cowpeas) in brown and vegetables (cow peas leaves and kale) in blue from Makueni County

The concentrations of F in the five food crop samples, as dry weight, are presented in Fig. 3. The order of concentrations increased from grains to vegetables. For the grains, maize had the lowest F concentration of 29 mg/kg, followed by cowpeas (36.6 mg/kg), while green grams had the highest value (71.2 mg/kg). F concentrations in the two vegetables ranged between 388 mg/kg in cowpeas leaves and 700 mg/kg in kale.

### F concentration in the farm soil

The water-soluble F concentration of 30 soil samples from farms in the area ranged from 0 to 3.47 mg/kg with a mean value of 0.87 mg/kg and a standard deviation of 0.98. This F concentration represent a fraction readily available for plant uptake (García & Borgnino, 2015). The mineralogical composition of the soil was analyzed to indicate the possible geogenic source of the water-soluble F observed in the farm soil in the area. The total semi-quantitative mineral compositions of the analyzed soil samples, their range, average, and standard error are shown in Table 1. According to Garcia and Borgnino (2015), apatite, biotite, and muscovite are the likely F-hosting minerals present in the soil. These authors reported that F occurs in mineral lattice in these minerals and will only be available for plant absorption upon weathering of these minerals. In the studied soil samples, these minerals (apatite, biotite, and muscovite) make up to 23% of the total mineral composition (Table 1), indicating that they are possible geogenic sources of F.

**Table 1:** The semi-quantitative abundance of minerals in 20 farm soil samples from Makueni County as obtained from XRD

Mineral group		Range (%)	Average (%)	Standard error
Plagioclase feldspars	Albite	1–20	10	1.24
	Anorthite	0–52	14	3.25
*K*-feldspars	Orthoclase	0–48	9	2.48
	Microcline	0–32	10	1.83
	sanidine	0–6	1	0.34
Micas	Muscovite	2–39	15	2
	Biotite	0–17	4	0.98
Quartz	Quartz	6–41	14	1.89
Clays	Dickite	0–9	2	0.65
	Kaolinite	0–8	2	0.63
	Illite	2–25	8	1.4
Apatite	Apatite	1–8	4	0.47
Pyroxenes	Enstatite	0–9	2	0.58
	Diopside	0–9	2	0.55
	augite	0–4	1	0.33
Oxides	Goethite	0–6	1	0.34
	Anatase	0–7	1	0.35

### F transfer factor (TF)

The concentrations of water-soluble F in the soils from the five farms where the analyzed food crops were collected are shown in Table 2. The transfer factor of F in all the analyzed food crops ranged from 26 to 257 (Table 2). There was a significant strong positive (*p* = 0.03, *r* = 0.89) correlation between the concentration of water-soluble F in farm soil and F concentration in food crops grown in those soils. A TF value greater than 1 indicates a high accumulation factor of F in the crops (Gupta & Banerjee, 2011).

### F exposure dose and health risk assessment

**Fig. 4:**
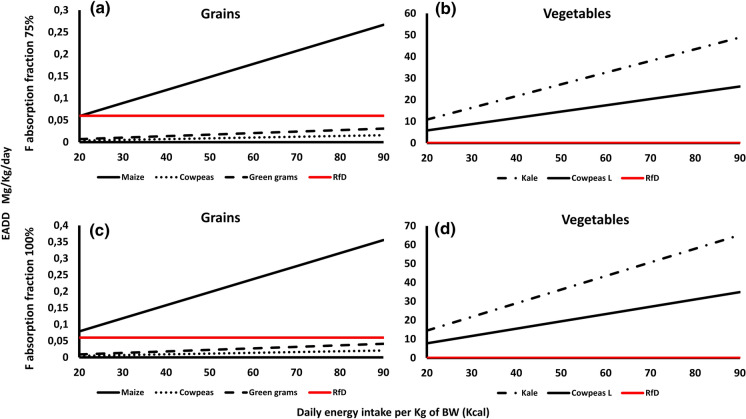
The EADD due to the consumption of the five analyzed food crops (raw) at different levels of daily energy intake per Kg of body weight from Makueni County. The reference dose (RfD), in red, indicates the no observable adverse effects level (NOAEL), the level below which F has on humans health effects (ATSDR, 2003). The F intake was considered at the two hypothetical absorption rates of 75% and 100% (Rizzu et al., 2020) in the top and bottom frames, respectively

i) Exposure dose in the raw food crops
  The estimated F average daily dose (EADD) of the analyzed food crop samples at the two hypothetical absorption limits of 75 and 100% against the daily calorie intake (DCI) range of 20 kcal for adults and 90 kcal for children are presented in Fig. 4. The doses were divided into grain-based foods (Fig. 2a and c) and vegetables (Fig. 2b and d). Among the grains, both cowpeas and green grams had lower EADD values than the recommended F RfD values of 0.06 mg/Kg at both hypothetical absorption rates. Cowpeas values ranged from 0.004–0.016 between 20 and 90 kcal DCI, whereas green grams values ranged from 0.007–0.03 between the two DCI values. In the 75% absorption rate, maize had a lower EADD value (0.05) compared to the F RfD in the 20-kcal energy intake, but it had a higher value of 0.27 in the 90 kcal energy intake. In the 100% absorption rate, maize had higher EADD values (0.08–0.36) than the RfD in both the 20 and 90 kcal energy intake range.
  Considering the two vegetables, both kale and cowpeas leaves had higher EADD values than the RfD of 0.06 mg/kg in both the 20 and 90 kcal energy intakes (Fig. 2b and 2d). In the 75% absorption rate, kale EADD values ranged from 10.86 to 48.87 while cowpeas leaves values ranged from 5.82 to 26.19. In the 100% absorption rate, kale EADD values ranged between 14.48 and 65.17 while cowpeas values ranged between 7.76 and 34.92.
ii) Exposure dose in cooked food

**Table 2:** Fluoride concentration in the five food crops, the five farm soil where they were grown, and their Transfer Factor (TF) from Makueni County

Sample	Maize (*Zea mays*)	Green grams (*Vigna radiata*)	Cowpeas (*Vigna unguiculata*)	Cowpeas leaves	Kale (*Brassica oleracea var. viridis*)
F concentration (mg/kg)	29	71.2	36.6	388	700
*F* concentration in soil (mg/kg)	1.1	0.97	1.52	1.52	2.72
TF	26	73	24	255	257

**Fig. 5:**
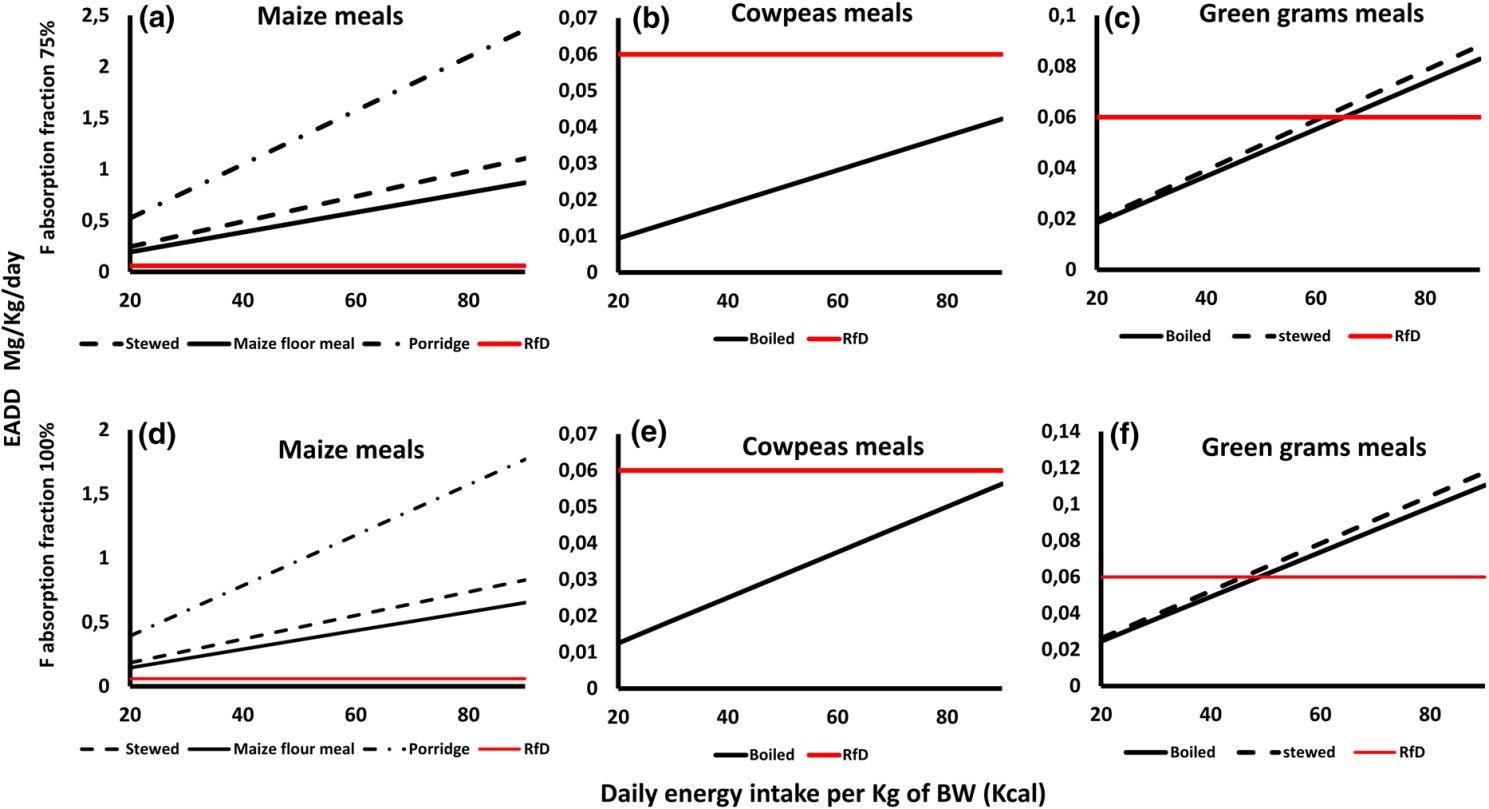
The EADD due to the consumption of different meals prepared from the three analyzed grain-based food crops at different levels of daily energy intake per Kg of body weight, at the two hypothetical absorption rates of 75% and 100% from Makueni County

We determined the hypothetical F dose in different meals prepared from the sampled and analyzed food crops as highlighted in the data analyzed section. The resultant EADD_F_ values calculated using the E values (FAO & Government of Kenya, 2018) of the various meals against the daily energy intake range of 20 kcal and 90 kcal are presented in Fig. 5.

The common maize-based foods whose E values were reported by the Government of Kenya (FAO & Government of Kenya, 2018) include stewed dry maize, maize floor meal (*ugali)*, and maize porridge. All the maize-based foods had higher EADD values than the RfD of 0.06 mg/kg within both the 20 kcal and 90 kcal energy intakes at both the 75 and 100% absorption rates (Fig. 5a and d). Maize porridge had the highest EADD values at both 75 and 100% absorption rates (0.39–1.77 and 0.52–2.36, respectively), followed by stewed dry maize (0.18–0.83 and 0.25–1.11, respectively), whereas maize floor meal (*ugali*) had the lowest values ranging (0.15–0.65 and 0.19–0.87, respectively).

The energy content (*E*) of dry boiled cowpeas was the only cowpeas meal reported. Within both the 75% and 100% absorption rates, the EADD values of boiled cowpeas were below the F RfD value of 0.06 mg/kg (Fig. 5b and e). The values were between 0.01–0.04 and 0.01–0.06 in the two absorption rates, respectively. The green grams meal used was boiled. There was a similar trend in EADD values in these two meals (Fig. 5c and f). At the 75% absorption rate, the EADD values ranged from 0.02 to 0.09, where values below the RfD value were between the 20 kcal and 60 kcal energy intakes. In the 100% absorption rate, the EADD values ranged between 0.02 and 0.12, where values below the RfD were observed between the 20 kcal and 50 kcal energy intakes (Table 2).

**Fig. 6:**
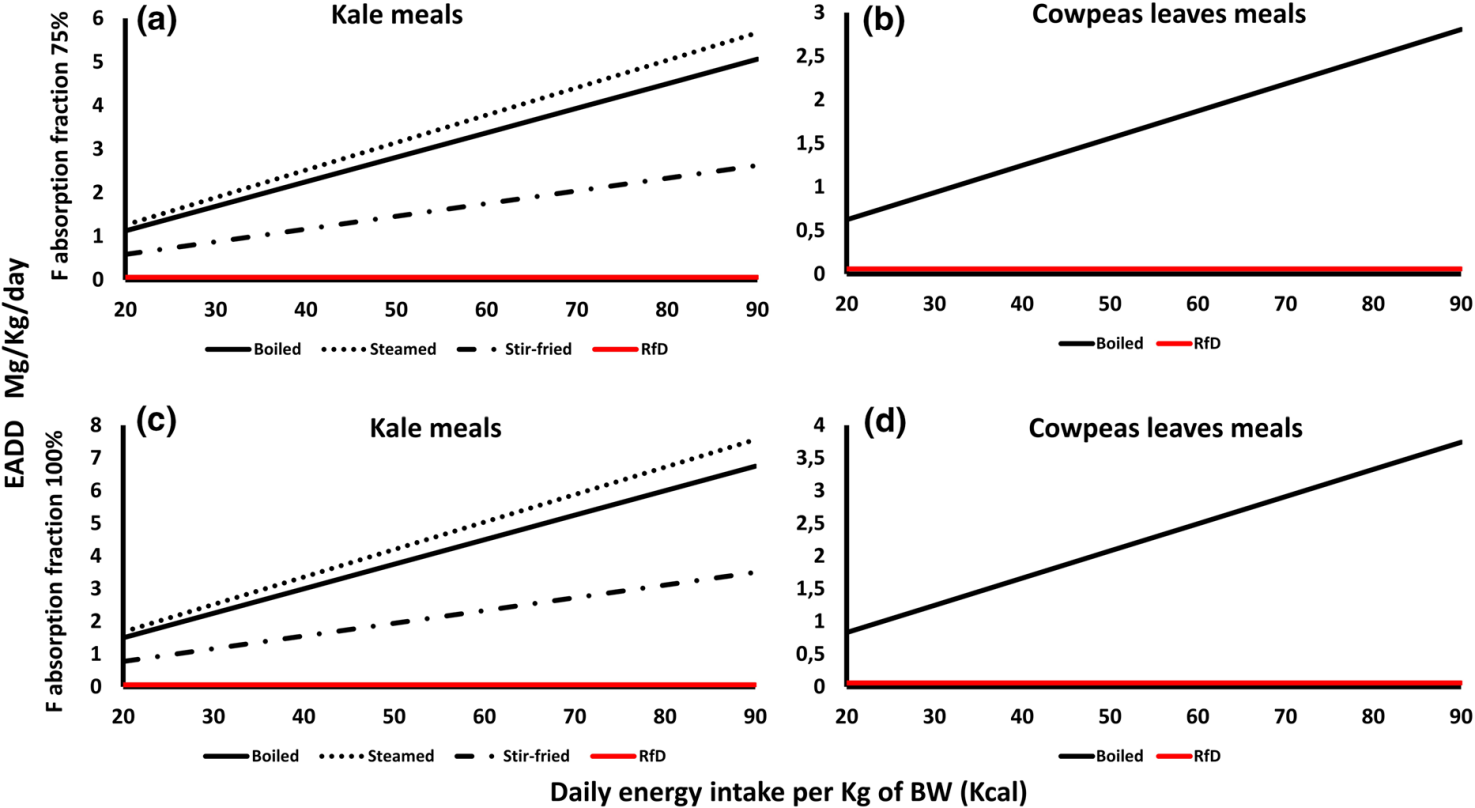
The EADD due to the consumption of different meals prepared from the two analyzed vegetables at different levels of daily energy intake per Kg of body weight, at the two hypothetical absorption rates of 75% and 100% from Makueni County

Among the vegetables, the *E* values of boiled, steamed, and stir-fried kale and boiled cowpeas leaves were reported (Fig. 6). All the kale meals had EADD values higher than the RfD of 0.06 mg/kg in both the 75 and 100% absorption rates (Fig. 6a and c). Steamed kale had the highest EADD values (1.26–5.67 and 1.68–7.56) in the two absorption rates followed by boiled (1.13–5.06 and 1.5–6.75) and stir-fried (0.58–2.63 and 0.78–3.5) meals. Similarly, boiled cowpeas leaves had higher EADD values than the RfD in both absorption rates (0.62–2.81 and 0.83–3.74) (Fig. 6b and d).

The Hazard Quotient (HQ) and cumulative hazard index (HI) of all the analyzed food samples (uncooked) are presented in Table 3. All the cumulative HI values between the two absorption rates (75 and 100%) were higher than the ‘no adverse effect’ level of 1. The cumulative HI values ranged between 279 and 1256 in the 75% F absorption rate and 372 and 1675 in the 100% F absorption rate. Kale and cowpeas leaves had the highest HQ values and thus were the major contributors to the high values of cumulative HI observed.

**Table 3:** The hazard quotient and cumulative hazard index of the five analyzed food crops (raw) at different levels of daily energy intake per Kg of body weight, at the two hypothetical absorption rates of 75% and 100% from Makueni County

Daily energy intake per Kg of BW	F absorption fraction 75%							F absorption fraction 100%					
	Hazard Quotient (HQ)												
	Maize	Cowpeas	Green grams	Kale	Cowpeas leaves	HI		Maize	Cowpeas	Green grams	Kale	Cowpeas leaves	HI
20	0.99	0.06	0.11	181	97	279		1.32	0.08	0.15	241	129	372
30	1.48	0.09	0.17	271	145	418		1.98	0.12	0.23	362	194	558
40	1.98	0.12	0.23	362	194	558		2.63	0.16	0.31	482	258	744
50	2.47	0.15	0.29	452	242	697		3.29	0.19	0.38	603	323	930
60	2.96	0.18	0.34	543	291	837		3.95	0.23	0.46	724	388	1116
70	3.46	0.2	0.4	633	339	977		4.61	0.27	0.53	844	452	1302
80	3.95	0.23	0.46	724	388	1116		5.27	0.31	0.61	965	517	1489
90	4.44	0.26	0.52	814	436	1256		5.93	0.35	0.69	1086	582	1675

## Discussion

### F concentrations in food crops

Vegetables (cowpeas leaves and kale) showed higher F concentrations (388 to 700 mg/kg) than the grain-based crops (29 mg/kg in maize to 71.2 mg/kg in green grams). These concentrations are also higher than those (7–215 mg/kg) reported in kale, cabbage, amaranth, pumpkin, and cowpeas leaves from other parts of Kenya (Owuor, 1985), as well as those reported in cabbage (296 mg/kg) from India (Begum et al., 2008; Gupta & Banerjee, 2011; Saxena & Sewak, 2015) and in curly kale, endive, and lettuce (40–146 mg/kg) from the Netherlands (Slooff et al., 2017).

## F concentration and sources in farm soil and transfer factor (TF)

The concentration of soil water-soluble F (up to 3.47 mg/kg) was higher than those reported in farm soils in Zaria, northern Nigeria (up to 0.2 mg/kg) (Okibe et al., 2010) and in Pennsylvania, USA (up to 1.5 mg/kg) (Gilpin & Johnson, 1980). However, higher values (up to 133.1 mg/Kg) were reported in farm soils in northern Tanzania (Rizzu et al., 2020).

The probable source of water-soluble F in farm soils in the study area is geogenic. This origin is supported by the presence of F-bearing minerals such as apatite, muscovite, and biotite, which make up to 23% of the soil mineralogical composition. This links to studies which consider biotite, muscovite, and apatite as the main sources of F in granitic regions based on the rate of weathering of these minerals (Currell et al., 2011; Edmunds & Smedley, 2013; García & Borgnino, 2015). Soil pH ranging between 3.8 and 7.2 was reported in farm soils in Makueni County (NAAIAP, & KARI, 2014), suggesting a relatively high F solubility potential in soils in the area (Ozsvath, 2009). The high F concentration in farm soil in the area is reflected in the TF values observed, which are higher in the vegetable samples (TF = 255–257) than in the grains (TF = 24–73). The significant positive correlation between the F concentration in food crops and farm soils suggests that farm soils in the study area contribute to the F absorbed by food crops. This relationship was also reported by several other studies (Li et al., 2017; Ruan & Wong, 2001; Wang et al., 2012).

In addition to uptake from soil, the high F concentration in the analyzed crops could also result from irrigation with high F water. A recent study by Gevera et al. (2020) showed that different groundwater sources, used for domestic and agricultural purposes, in the study area were enriched in F (of up to 7.17 mg/l). Additionally, the farms where kale and cowpeas samples were collected, use borehole water with high F concentrations (between 5.19 and 7.17 mg/l) for irrigation. F from irrigation water can be absorbed into crops directly through leaves stomata and root uptake (Rizzu et al., 2021).

## F exposure and health risk assessment

The calculated EADD and HI values suggest that the analyzed food crops considerably contribute to the daily F intake of the local population of the study area which may negatively impact their health. The EADD values of the grain-based crops (in both hypothetical 75% and 100% F absorption rates) were higher than the F reference dose for infants and children compared to adults. Vegetables had very high EADD values, well above the F reference dose for both adults and children in both hypothetical F absorption rates. This indicates that, children in the study area have a high risk of F related diseases from consuming both grain-based and vegetables, whereas adults are more vulnerable if they follow a largely vegetable diet.

The vulnerability of children to higher dietary F intake than adults was also reported in northern Tanzania (Rizzu et al., 2020), eastern India (Bhattacharya et al., 2017), and southern Pakistan (Kazi et al., 2019). This was associated with a higher intestinal absorption rate and higher energy requirements relative to lower body weight in children compared to adults (Bhattacharya et al., 2017; Kazi et al., 2019; Rizzu et al., 2020; Stellman, 1998). Additionally, factors such as genetic determinants, sex, health status, dietary habits, substance abuse, physical fitness status, and concomitant exposure to other chemicals can influence the health implications of high F in diet in the area (Stellman, 1998).

When considering F dose from different meals prepared, all vegetable meals had high EADD values compared to the F reference dose for both adults and children while maize meals showed the highest EADD contribution among the grains. It should be noted that, the energy (*E*) value is what differs between these meals (and therefore impacts the F EADD value) and the preparation methods do not add extra F in the meals. However, F can be present in the cooking material and dust.

The high EADD values in maize porridge and maize flour meals, which are among the most consumed meals in Makueni County and across Kenya, indicate that they contribute significantly to the daily F intake. Boiled cowpeas had no F health risks indicating a potentially desirable target grain-based crop in the region. Kale is a common vegetable component of many Kenyan meals and showed the highest EADD values in the steamed meal while stir-fried kale had the lowest value. Clearly cooking method makes a significant difference on EADD. The HI values of all the analyzed food crops were greater than 1 indicating that all the food crops analyzed pose non-carcinogenic health effects to the local population. The high prevalence dental fluorosis affecting the local population (Gevera et al., 2020), attests to the link between F availability and health impacts. Similarly, when a health survey was conducted to determine the health effects of high F and salinity in drinking water and public awareness in the area, 91% of the participants reported knowing at least one person with dental fluorosis in their village (Gevera et al., 2022, under review).

## Concluding remarks and recommendations

The findings of this study show that vegetables (cowpeas leaves and kale) have higher F concentrations (388 and 700 mg/kg) than the grain-based crops (maize, cowpeas, and green grams) (29 to 71.2 mg/kg). The water-soluble F concentrations in the 30 samples of farm soil ranged between 0 to 3.47 mg/kg. One of the probable sources of F in the soil was geogenic, hosted by *F*-bearing minerals such as apatite, muscovite, and biotite present in the farm soils. The EADD analysis in the food samples indicated a potential F health risk mostly from consumption of the vegetables and maize meals. Children were at a higher risk of chronic F exposure in all the food samples due to their high daily energy requirement (high metabolic rate) per body weight. In addition, the use of high F irrigation water can contribute to the elevated F observed in the food crops. This causes a food security problem in the area that already has a constrained agricultural output due to semi-aridity. Therefore, besides the health risks associated with drinking of high F water in Makueni County, some food crops grown and consumed in the area could also contribute to the total daily F intake in a substantial way. In addition, different meals prepared from these food crops can also influence the level of F intake.

Based on these results, consideration should be given to the type of crops grown and consumed in the area. For example, grain-based crops, such as cowpeas legumes, with no F health risk should be encouraged for farming instead of green grams. Similarly, the types of meals prepared for the different age groups should be considered as evidenced by the high F exposure risk in maize porridge, which is a common meal for infants and children who are the most common vulnerable group in the area. Additionally, the use of high F water for irrigation should be discouraged in the area due to its potential contribution to F uptake by crops. Finally, further research is required to determine the amount of F in food consumed in the area as well as the dietary habits of the local population to determine the total F intake, which will help in establishing F risk factor in the area.

## Data Availability

The authors confirm that the data generated during this study are presented in the manuscript. However, additional data generated can be available upon request from the corresponding author (P. Gevera).
